# Task-Based Cognitive Fatigability for Older Adults and Validation of Mental Fatigability Subscore of Pittsburgh Fatigability Scale

**DOI:** 10.3389/fnagi.2018.00327

**Published:** 2018-10-19

**Authors:** Sarah E. Burke, Immanuel Babu Henry Samuel, Qing Zhao, Jackson Cagle, Ronald A. Cohen, Benzi Kluger, Mingzhou Ding

**Affiliations:** ^1^Department of Neuroscience, College of Medicine, University of Florida, Gainesville, FL, United States; ^2^J. Crayton Pruitt Family Department of Biomedical Engineering, Herbert Wertheim College of Engineering, University of Florida, Gainesville, FL, United States; ^3^Department of Clinical and Health Psychology, College of Public Health and Health Professions, University of Florida, Gainesville, FL, United States; ^4^Departments of Neurology and Psychiatry, Anschutz School of Medicine, University of Colorado, Aurora, CO, United States

**Keywords:** cognitive fatigability, Stroop task, fatigue, performance measures, validation

## Abstract

Cognitive fatigue and cognitive fatigability are distinct constructs. Cognitive fatigue reflects perception of cognitive fatigue outside of the context of activity level and duration and can be reliably assessed via established instruments such as the Fatigue Severity Scale (FSS) and the Modified Fatigue Impact Scale (MFIS). In contrast, cognitive fatigability reflects change in fatigue levels quantified within the context of the level and duration of cognitive activity, and currently there are no reliable measures of cognitive fatigability. A recently published scale, the Pittsburgh Fatigability Scale (PFS), attempts to remedy this problem with a focus on the aged population. While the physical fatigability subscore of PFS has been validated using physical activity derived measures, the mental fatigability subscore of PFS remains to be tested against equivalent measures derived from cognitive activities. To this end, we recruited 35 older, healthy adult participants (mean age 73.77 ± 5.9) to complete the PFS as well as a prolonged continuous performance of a Stroop task (>2 h). Task-based assessments included time-on-task changes in self-reported fatigue scores (every 20 min), reaction time, and pupil diameter. Defining subjective fatigability, behavioral fatigability, and physiologic/autonomic fatigability to be the slope of change over time-on-task in the above three assessed variables, we found that the PFS mental subscore was not correlated with any of the three task-based fatigability measures. Instead, the PFS mental subscore was correlated with trait level fatigue measures FSS (ρ = 0.63, *p* < 0.001), and MFIS cognitive subsection (ρ = 0.36, *p* = 0.03). This finding suggested that the PFS mental fatigability subscore may not be an adequate measure of how fatigued one becomes after a given amount of mental work. Further development efforts are needed to create a self-report scale that reliably captures cognitive fatigability in older adults.

## Introduction

Fatigue is a common complaint in older adults and is associated with poor quality of life, functional disability, and increased mortality (Cathébras et al., 1992; Gill et al., 2001; Hardy and Studenski, 2008a,b). Despite the apparent importance of fatigue as a public health concern, research in this area has long been hampered by problems associated with definition and measurement (Muscio, 1921). To address this issue, recent work has suggested a unified taxonomy for more precisely communicating and describing the construct of fatigue (Kluger et al., 2013). Important aspects of this taxonomy include: (1) Distinguishing subjective fatigue (feelings of lack of energy or increased effort) from objective performance fatigability (changes in performance over time); (2) distinguishing fatigue from related phenomena (e.g., depression and sleepiness); (3) specifying what domains of performance are affected by fatigue (e.g., cognitive or physical); and (4) describing what physiologic factors are associated with fatigue.

The concept of fatigability, reflecting the change in self-reported fatigue or behavior as a result of a given activity, is particularly relevant to older adults as highly fatigable individuals may limit their activities in an effort to reduce subjective fatigue (Eldadah, 2010). Given the well-documented adverse consequences of limiting one’s activities, there is thus a great need to develop and validate measures of both performance and subjective fatigability, which are essential for diagnosis and treatment.

The Pittsburgh Fatigability Scale (PFS) represents recent efforts to meet this need. Its *physical fatigability* subscore demonstrated good concurrent and convergent validity against physical performance-based measures of behavioral and perceived physical fatigability for the aging population (Glynn et al., 2015). Its *mental fatigabilit*y subscore, however, has not been validated against any cognitive performance-based metrics. The goal of the current study was thus to assess the construct validity of the PFS mental fatigability subscore by determining whether it was associated with task-based subjective, behavioral, or physiological fatigabilities. Here, subjective fatigability was measured by the rate of change of serial self-reported fatigue scores during the prolonged performance of a cognitive task; behavioral fatigability was measured by the rate of change in reaction times over time-on-task; physiologic/autonomic fatigability was measured by the rate of change in pupil diameter as a result of participants fatiguing (Hopstaken et al., 2015).

## Materials and Methods

### Participants

Thirty-five older adults (>60 years of age) were recruited through newspaper advertisements and flyers. Participants were compensated for their time with a $50 gift card for each visit. The Fatigue Severity Scale (FSS) (Krupp et al., 1989) score was collected during the initial phone screening. Participants were eligible for the study if they were free from diagnoses of either neurological disorders or non-neurological disorders that might contribute to fatigability, including the following: cardiac, respiratory, endocrine disorders; currently receiving treatment for cancer; severe depression; attention deficit disorder; or sleep disorders. All participants were native English speakers and were free from any reading, hearing, or vision impairments.

### Baseline Assessments

Participants were tested for subjective, objective, and behavioral dimensions of fatigue and fatigability over the course of two visits. For the first visit, all participants completed the PFS, a 10-item scale in which they were asked to rate the imagined fatigue level, from a score of 0 (no fatigue) to 5 (extreme fatigue), that would arise from participation in activities of specific duration and intensity (Glynn et al., 2015). Mental and physical subscores were summed separately. Participants were also asked to complete other questionnaires (**Table 1**) including the FSS, a 9-item scale to assess degree of fatigue symptoms, the Modified Fatigue Impact Scale (MFIS) (Fisk et al., 1994), a 21-item scale to assess the effects of fatigue on cognition, physical activity, and psychosocial functioning, the 36-Item Short Form Survey (SF36) (Ware and Sherbourne, 1992) measuring general well-being, the Hospital Anxiety and Depression Scale (HADS) measuring depression symptoms, the Pittsburgh Sleep Quality Index (PSQI) (Buysse et al., 1989) measuring sleep quality, and the Epworth Sleepiness Scale (ESS) (Johns, 1991) measuring sleep quality. Cognitively, participants were given a brief test of cognitive function, the Montreal Cognitive Assessment (MOCA) (Nasreddine et al., 2005), and a computerized test of executive working memory function, the Operation Span task (OSPAN) (Unsworth et al., 2005).

**Table 1:** Measures collected on the first visit (baseline).

Instruments	Domain assessed
FSS (Krupp et al., 1989)	Fatigue
Modified fatigue impact scale (MFIS) (Fisk et al., 1994)	Fatigue
36-Item short form survey (SF36) (Ware and Sherbourne, 1992)	General health
Pittsburg sleep quality index (PSQI) (Buysse et al., 1989)	Sleep quality
The epworth sleepiness scale ESS (Johns, 1991)	Sleepiness
Hospital anxiety and depression scale (HADS) (Zigmond and Snaith, 1983)	Depression
Pittsburgh sleep quality index (PSQI) (Buysse et al., 1989)	Sleep amount
Montreal cognitive assessment (MOCA) (Nasreddine et al., 2005)	Cognition
The operation span task (OSPAN) (Unsworth et al., 2005)	Working memory

### Cognitive Fatigability Task

**FIGURE 1:**
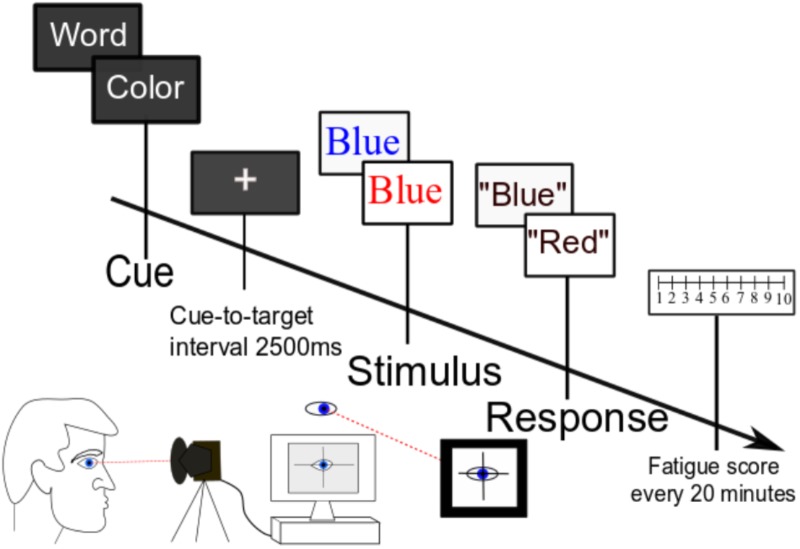
Task paradigm. Participants performed a cognitively demanding cued Stroop task for 2.5 h without break. Serial subjective fatigue scores, reaction time data, and pupil diameter were recorded continuously throughout the experiment.

During the second visit, participants completed a cued Stroop task for a sustained 160 min without break (**Figure 1**). Every 20 min during the task, participants were presented with a fatigue scale and asked to rate the current fatigue level from 1 to 10. Five participants were excluded from the analysis as they asked to quit within 1 h of task start. One additional participant was excluded from the reaction time analysis for data recording issues.

As shown in **Figure 1**, at the beginning of each trial, participants were presented with a cue of either “color” (50% of trials) or “word” (50% of trials). Following a 2500-ms cue-to-target interval, they were then presented with a target word in either a congruent font (the color of the font matches the meaning of the written word) or an incongruent font (the font color does not match the meaning of the word). If the trial is cued for “color,” the subject’s task was to name the color of the font and disregard the meaning of the written word; if the trial is cued for “word,” the subject’s task was to read the word and ignore the font color. The next trial started when the experimenter recorded the response uttered by the participant. We chose the Stroop task because cognitive fatigue is associated with impairment of executive function (van der Linden et al., 2003), and this task stresses executive control over conflict processing (Jensen and Rohwer, 1966). Further, the Stroop task has been shown to reliably induce fatigue in our prior studies of young participants (Wang et al., 2014).

The Stroop task was programmed in the Presentation software. Pupil diameter was monitored using an Eye Tracker (SR Research EyeLink 1000), which was synced to the Presentation software. Continuous pupil data was sampled at 1000 Hz and epoched from -200 ms to 3500 ms for each trial. We were mainly interested in changes in tonic pupil diameter rather than stimulus-related changes in pupil diameter. Thus, pupil diameter in the precue time period (-200 ms to 0 ms), normalized to the baseline condition at the beginning of the task, was analyzed for time-on-task changes. Four participants were excluded from pupillometry analysis for excessive head movements. To minimize variability, all participants were coached on the behavioral task by one designated experimenter with a uniform script and tested at 9:00 AM. They were asked to get a usual night’s sleep prior to the visit and verified that they were able to do so on the morning of the task. They were also asked to avoid caffeine on the day of testing. Participants were provided with a chin rest to ensure that the head stayed in the same position throughout the task. They were also provided with a pillow for back support to help keep the upper body stationary. Further, they were reminded to keep head movements to a minimum during trials and to keep their gaze and head position in line toward a continuous central fixation point on the screen.

Task-based fatigability was defined in several domains by the rate of change of the pertinent variables. Subjective fatigability was calculated to be the slope of the fatigue scores over the course of the experiment (rate of change). The slope of reaction time change over the experiment duration was calculated as a measure of behavioral fatigability. Physiologic/autonomic fatigability was calculated as the rate of change in pupil diameter.

### Statistical Analysis

Error trials, trials with reaction time less than 500 ms or longer than 4000 ms were excluded from analysis. A mixed linear model for repeated measures was applied to fatigue rating, reaction time, and pupilometry data. Survey and scale data were collected via Redcap software package and analyzed in MATLAB. Relations among survey/scale data and behavioral, perceptive, and autonomic fatigability indices were analyzed using Spearman’s rank correlation, controlling for the effect of age and depression (HADS). Multiple comparisons were corrected with Bonferroni–Holm (Holm, 1979) correction at the alpha value of 0.05.

## Results

Thirty-five older adults (14 male; 40%), mean age 73.77 ± 5.90 with a range of 63–84, participated in the study. Trait fatigue levels, based on the FSS (Krupp et al., 1989), ranged from 1.11–6.44 with mean score of 3.94 ± 1.53. PFS mental subscores had a mean of 14.00 ± 9.82 and ranged from 0 to 35. Scores did not differ between gender (*p* = 0.98), with mean score being 14.29 ± 8.42 for male and 13.81 ± 9.36 for female, and there was no effect of age (*p* = 0.67).

### Relation Between PFS Mental Fatigability Subscores and Other Self-Report Measures

**FIGURE 2:**
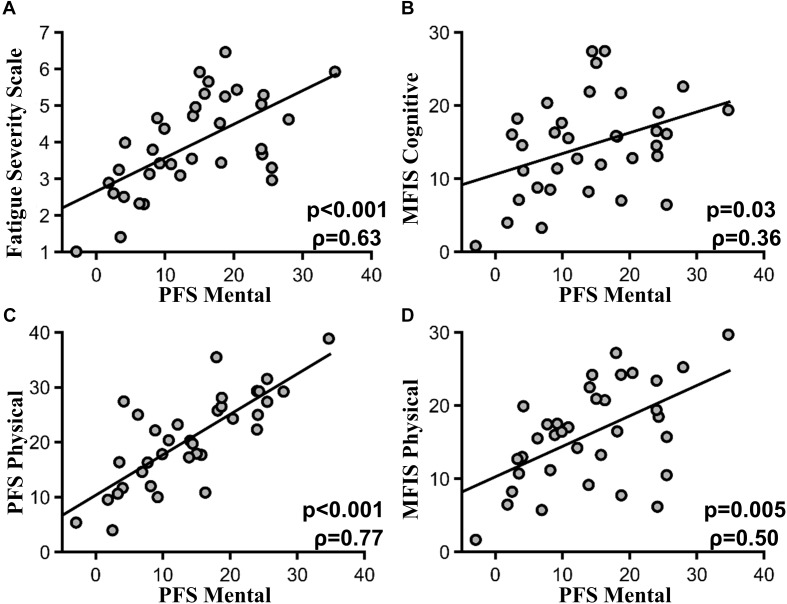
PFS mental subscore and other fatigue scales. **(A)** Pittsburgh mental fatigability subscores and FSS (*p* < 0.001, ρ = 0.63). **(B)** Pittsburgh mental fatigability subscores and MFIS cognitive subscore (*p* = 0.03, ρ = 0.36). **(C)** PFS mental and physical subscore (*p* < 0.001, ρ = 0.77). **(D)** PFS mental subscore and MFIS Physical subscore (*p* = 0.005, ρ = 50).

PFS mental fatigability subscores were highly correlated with the FSS scores (ρ = 0.63, *p* < 0.001) (**Figure 2A**). PFS mental fatigability subscores were also correlated with the MFIS cognitive subscore (ρ = 0.36, *p* = 0.03) (**Figure 2B**). In addition, PFS mental fatigability subscores were correlated with PFS physical fatigability subscore (ρ = 0.77, *p* < 0.001) (**Figure 2C**), as well as MFIS physical subsection score (ρ = 0.50, *p* = 0.005) (**Figure 2D**). However, no correlations (or weak correlations) were found with the baseline health surveys such as SF36 general health questionnaire (ρ = -0.37, *p* = 0.11) or depression scale (HADS) (ρ = 0.41, *p* = 0.09), or with the sleep surveys such as ESS (ρ = .41, *p* = 0.09) or PSQI (ρ = 0.29, *p* = 0.26). There was no correlation between PFS mental fatigability subscore and the MOCA score (ρ = -0.05, *p* = 0.80) and the OSPAN score (ρ = -0.20, *p* = 0.52).

### Relation Between PFS Mental Fatigability Subscore and Task-Based Subjective Fatigability

**FIGURE 3:**
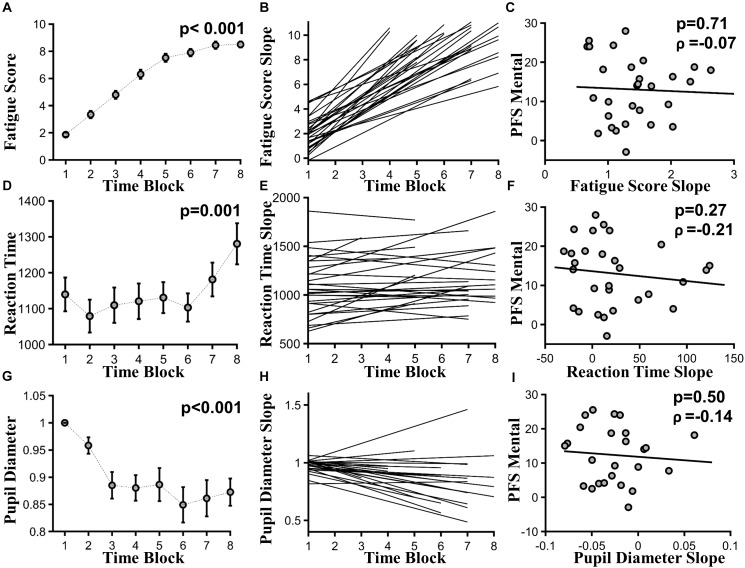
Task-based fatigability. **(A)** Group mean subjective fatigue score by 20-min time blocks, *F*_(8,325)_ = 5.83, *p* < 0.001, effect size > 3. **(B)** Linear fits to individual subjective fatigue scores as function of time-on-task. **(C)** Subjective fatigability [slopes of the linear fits in **(B)**] versus PFS mental subscores (ρ = –0.07, *p* = 0.71). **(D)** Group mean RT by 20-min time blocks, *F*_(7,169)_ = 3.71, *p* = 0.001, effect size = 0.24. **(E)** Linear fits to individual reaction time as function of time-on-task. **(F)** Behavioral fatigability [slopes of the linear fits in **(E)**] versus PFS mental subscores (ρ = -0.21, *p* = 0.27). **(G)** Group mean pupil diameter by 20-min time blocks, *F*_(7,134)_ = 6.86, *p* < 0.001, effect size = 1.33. **(H)** Linear fits to individual pupil diameter as function of time-on-task. **(I)** Autonomic fatigability [slopes of the linear fits in **(H)**] versus PFS mental subscores (ρ = –0.14, *p* = 0.50).

As time-on-task progressed, subjective fatigue ratings increased significantly, as assessed across 20-min time blocks, where *F*_(8,325)_= 5.83, *p <* 0.001, and effect size > 3 (**Figure 3A**). At the individual subject level, linear fit to subjective fatigue scores as a function of time-on-task was calculated, and the slope was used as a measure of subjective fatigability (**Figure 3B**). PFS mental fatigability subscore and subjective fatigability were not significantly correlated (ρ = -0.07, *p* = 0.71) (**Figure 3C**).

### Relation Between PFS Mental Fatigability Subscore and Task-Based Behavioral Fatigability

As time-on-task progressed, RT slowed from the first 20-min time block to the last, where *F*_(7,169)_ = 3.71, *p* = 0.001, and effect size = 0.24 (**Figure 3D**). Accuracy did not decline significantly, where *F*_(7,160)_ = 0.691, *p* = 0.68, and effect size = 0.39. At the individual subject level, linear fit to RT measured in 20-min blocks was calculated and shown in **Figure 3E**. Slopes of the linear fits were defined as behavioral fatigability. There was no correlation between behavioral fatigability and PFS mental fatigability subscore (ρ = -0.21, *p* = 0.27) (**Figure 3F**).

### Relation Between PFS Mental Fatigability Subscore and Task-Based Autonomic Fatigability

As time-on-task progressed, pupil diameter decreased significantly across 20-min time blocks, where *F*_(7,134)_ = 6.86, *p* < 0.001, and effect size = 1.33 (**Figure 3G**). At individual subject level, linear fit to pupil diameters measured in 20-min blocks was calculated, and shown in **Figure 3H**. Autonomic fatigability, defined as the slopes of the linear fits, did not correlate with PFS mental fatigability subscore (ρ = -0.14, *p* = 0.50) (**Figure 3I**).

## Discussion

We attempted to validate the PFS mental fatigability subsection using task-based fatigabilities. Prolonged performance of the cued Stroop task induced cognitive fatigue. As time-on-task progressed, subjective fatigue scores increased, reaction time slowed, and pupil diameter decreased. Accuracy, however, remained stable throughout the task. It is possible that a speed-accuracy tradeoff took place because it is known that older adults have the tendency to trade speed in favor of higher accuracy (Salthouse, 1979; Forstmann et al., 2011). Individual slopes of the linear fits to these measures as function of time-on-task were defined as task-based fatigabilities. The main finding of this work is that scores on the mental subsection of the PFS did not correlate with any of the three task-based fatigabilities. In contrast, PFS mental fatigability subscores were highly correlated with other instruments that measure cognitive fatigue (the FSS and the MFIS) and physical fatigue/fatigability (subsections of the PFS and of the MFIS) outside of the context of specifically defined activity amount and duration. The lack of correlation between general fatigue survey measures and task-based self-reports has been found before (Sandry et al., 2014). To our knowledge, however, this study is the first to test a questionnaire based measure of mental fatigability against task-based cognitive fatigabilities.

How to explain the lack of relation between PFS mental subscore and task-based fatigability? A potential issue in the PFS that may be hindering efficacy is that physical and cognitive fatigability are often confused for each other in common vernacular because research to date has not produced consistent nomenclature (Chaudhuri and Behan, 2004; Kluger et al., 2013). Therefore, participants may have encountered conflicting terms from professionals when referencing physical and cognitive domains. Specifically, in the PFS survey, participants are asked to rate the imagined fatigue levels for a given activity without actually going through the activity. They are asked to rate physical fatigability and mental fatigability individually based on the one activity, which has both physical and mental dimensions. In our data, the physical and cognitive PFS subscores are highly correlated, suggesting that participants may be conflating the two when rating the physical and cognitive fatigability levels associated with the imagined example activity.

Another issue could be that proper assessment of cognitive fatigability requires a clear understanding of cognitive effort (Boksem and Tops, 2008) within a specific, quantifiable amount of activity (Eldadah, 2010). In the cognitive domain, perceived effort is influenced by motivation (van der Linden, 2011; Hopstaken et al., 2016) that is itself affected by the level of task enjoyment (Puca and Schmalt, 1999; Boksem and Tops, 2008). These factors are highly influential in perception of effort but have been hard to define in the cognitive domain; there is not yet a consensus in the literature as to the proper definition (van der Linden, 2011). In the PFS, the example activities are very specific and quantifiable with respect to physical effort for a task, such as sitting watching television for an hour. However, the amount of mental effort required for this activity may vary based on the type of program that is being watched or similar factors that impact how enjoyable it is to the individual. Therefore, the scale may benefit from using cognitive tasks that minimize the influence of personal preference. Since motivation is a very important factor in fatigue (Boksem et al., 2006; Gergelyfi et al., 2015; Hopstaken et al., 2016), it should be better qualified within questions that ask an individual to rate a task. For example, the questions on the PFS might clarify activities that a task is one that “you find enjoyable” or that “you consider to be a chore” and provide several examples within a similar range of intensity. With better characterization of the type of activity that is being asked about, the effect of motivation can be taken into account rather than varying between individuals based on interpretation. The authors of the PFS do a very good job of making these kinds of specifications for the more physically oriented activities, but the cognitively oriented activities are more multifaceted, and thus more difficult to specify.

The study’s limitations include that the Stroop task does not impose time restrictions on responses within a trial; self-pace may reduce the reliability of objective measures (reaction time and pupil diameter). Also, behavioral fatigability measures have been tightly linked to confounding factors, such as motivation (Hopstaken et al., 2015) or compensation (Christodoulou, 2005; Wang et al., 2014), so may be more difficult to directly interpret than subjective fatigability. Finally, pupil data should be interpreted with caution as it reflects the collective contribution of numerous brain systems and structures (Mathôt, 2018).

## Conclusion

The lack of correlation between task-based fatigability measures and the PFS mental subscore indicate that mental fatigability, as a more multifaceted construct, is difficult to capture using questions asking about fatigue as a result of previous or imagined experiences. Therefore, more detailed and specific questions that take into account factors such as motivation and preference may be necessary to make this scale a viable clinical tool.

## Ethics Statement

This study was carried out in accordance with the recommendations of the NIH Protection of Human Research Subjects and HIPPA for research guidelines with written informed consent from all subjects. All subjects gave written informed consent in accordance with the Declaration of Helsinki. The protocol was approved by the University of Florida Gainesville Health Science Center Institutional Review Board.

## Author Contributions

SB did conception, design, recruitment, data collection, data analysis, data interpretation, and manuscript preparation. IBHS did conception, design, data collection, data analysis, data interpretation, and manuscript preparation. QZ and JC did data analysis. RC did data interpretation and manuscript preparation. BK and MD did conception, design, data interpretation, and manuscript preparation.

## Conflict of Interest Statement

The authors declare that the research was conducted in the absence of any commercial or financial relationships that could be construed as a potential conflict of interest. The handling Editor declared a shared affiliation, though no other collaboration, with several of the authors SB, IBHS, QZ, JC, RC, and MD at the time of review.
